# Finger trocar: a safe method for entering the peritoneal cavity during laparoscopy in obese patients

**DOI:** 10.1308/003588412X13373405386015k

**Published:** 2012-09

**Authors:** R Durai, T Oke, M Siddiqui

**Affiliations:** South London Healthcare NHS Trust,UK

## BACKGROUND

We report an easy way of entering the peritoneal cavity in obese patients during aparoscopic surgery, which can be challenging.

## TECHNIQUE

A transverse incision is made five finger breadths away from the xiphoid process in the midline. The incision is deepened until the anterior rectus sheath is located. A 10mm transverse incision is made on the anterior rectus sheath. Using the surgeon’s finger in a rotating and dissecting manner as a trocar, the peritoneal cavity is entered. Then a 10mm non-bladed trocar port is inserted into the peritoneal cavity and pneumoperitoneum is created. Once the procedure is completed, the anterior rectus sheath defect is closed with three size 0 polyglactin sutures on a J needle and the skin is closed.

## DISCUSSION

In this method, finger tactile sensation guides entry into the peritoneal cavity, minimising injury to internal organs. This is lacking in the standard laparoscopic visual pneumoperitoneum creation techniques. Our method is safe and quicker than other methods. If the anterior rectus sheath incision is >10mm, the wound can leak gas. This can be minimised using a balloon or gel port. The incision on the rectus sheath should be precisely in the midline. Otherwise one will find the thick rectus muscle and posterior rectus sheath. These may provide resistance and a risk of bleeding. When the posterior sheath is difficult to enter, an artery clip may be used carefully along the side of your finger to make a tiny hole or to provide countertraction by grasping it. An opening can then be created safely in the posterior sheath/peritoneum.

